# Evaluating an enhanced adherence intervention among HIV positive adolescents failing atazanavir/ritonavir-based second line antiretroviral treatment at a public health clinic

**DOI:** 10.5897/JAHR2016.0406

**Published:** 2017-01-31

**Authors:** Tariro Dianah Chawana, David Katzenstein, Kusum Nathoo, Bernard Ngara, Charles Fungai Brian Nhachi

**Affiliations:** 1 Department of Clinical Pharmacology, University of Zimbabwe, Harare 00263, Zimbabwe; 2 Department of Medicine, Division of Infectious Diseases, Stanford University, California.; 3 Department of Paediatrics, University of Zimbabwe, Harare 00263, Zimbabwe.; 4 Department of Community Medicine, University of Zimbabwe, Harare 00263, Zimbabwe.

**Keywords:** Adolescents, HIV, second-line treatment failure, adherence, resistance

## Abstract

Sustaining virological suppression among HIV-infected adolescents is challenging. We evaluated a home-based adherence intervention and characterized self-reported adherence, virological response and drug resistance among adolescents failing atazanavir/ritonavir (ATV/r)-based 2^nd^ line treatment.

**Methods:**

HIV-positive adolescents (10–18 years) on ATV/r-based 2^nd^ line treatment with virological failure (viral load (VL) ≥1 000 copies/ml) were randomized to either standard care (SC) or SC with addition of modified directly administered antiretroviral therapy (mDAART) for 90 days. VL was measured and questionnaires were administered at study entry and at 3 months. Genotyping was done for participants with continued failure. Primary outcome was suppression to VL < 1 000 copies/ml.

**Results:**

Fifty adolescents aged 10–18 years on 2^nd^ line treatment for >180 days were enrolled, 23(46%) were randomized to mDAART and 27(54%) to SC. Fifty-four percent were female; mean age was 15.8 years; mean baseline VL was 4.8(log_10_) copies/ml; 40% reported adherence <80% in previous 1 month at baseline; 40% suppressed (VL <1 000 copies/ml) after follow-up. mDAART resulted in significantly increased self-reported adherence (RR= 0.1; 95% CI=0.02–0.8, p=0.023); closely following dosing schedule (RR= 4.8; 95% CI=1.6–13.8, p=0.004); VL decrease (p=0.031) and modest increase in virological suppression to <1 000 copies/ml (p=0.105). Genotyping in 28/30 participants with continued virological failure demonstrated high level atazanavir resistance (I50L, N88S and I84V) in 6(21%); 3(11%) of whom also had high level resistance to lopinavir and darunavir (V32I, I50L, I54V, 147V and V82A).

**Discussion:**

The mDAART intervention modestly improved virological suppression among adolescents with ATV/r-based 2^nd^ line treatment failure, significantly increased self-reported adherence and decreased viral load. High level ATV/r resistance was demonstrated.

**Conclusion:**

Targeting mDAART to adolescents who are virologically failing PI-based 2^nd^ line treatment decreases viral load and increases self-reported adherence. Early drug-resistance testing could reduce morbidity and mortality.

## INTRODUCTION

Global scale-up of antiretroviral therapy (ART) has significantly reduced HIV-related morbidity and mortality. However, sub-Saharan Africa (SSA) continues to bear the highest burden of HIV infection in the world, accounting for about 90% of all HIV infections (World Health Organisation, 2014). About 2.1 million adolescents (10–19 years of age) in 2012 were living with HIV globally (Lowenthal et al., 2014; WHO, UNAIDS, UNICEF, 2011). Over 10,000 HIV-infected adolescents were registered in HIV-care services in 2008 in Zimbabwe (Ferrand et al., 2010). Adolescents present important challenges to access, adherence and retention in care. Literature reports that 20 to 50% of HIV-infected adolescents on 2^nd^ line are failing treatment (Nglazi et al., 2012; Suaysod et al., 2015). Adolescents who fail boosted protease inhibitor (PI)-based 2^nd^ line regimens in resource-limited settings (RLS) have limited treatment options for salvage therapy, poor treatment outcomes, pose a risk of transmitting drug resistant virus and are at higher risk of subsequent treatment failure (Gupta et al., 2012; Hosseinipour et al., 2013).

Virological failure in adolescents is thought to be a result of poor adherence (Garone et al., 2014; Lessells et al., 2013; Levison et al., 2012). Paterson reported that >95% adherence is required for viral suppression on non-nucleotide reverse transcriptase inhibitors (NNRTIs) and boosted bPIs (Paterson et al., 2000). However, Kobin and Shutter later argued that for patients on boosted PIs, adherence rates of at least 80% are required for a minimum of 80% of patients to achieve viral suppression and that mean adherence required for viral suppression is 75% (Roux et al., 2011; Shuter et al., 2007; Shuter, 2008). Boosted PIs are therefore more ‘forgiving’ than NNRTIs.

Drug resistance could also cause 2^nd^ line treatment failure. Poor adherence selects drug resistance mutations due to on-going viral replication at sub-inhibitory PI concentrations (Nachega et al., 2009). However, boosted PIs have high genetic barrier to resistance, typically requiring multiple mutations, rather than single point mutations, for clinically significant drug resistance (Rhee et al., 2015; Tang and Shafer, 2012). Many studies of boosted PIs have noted the absence PI resistance in patients failing PI-based 2^nd^ line treatment (Garone et al., 2014; Levison et al., 2012).

The reasons why a high proportion of adolescents may fail boosted PI based 2^nd^ line treatment include poor adherence and evolution of drug resistance. If sub-optimal adherence is the reason, intensive adherence interventions should result in viral suppression. If drug resistance is the cause of treatment failure, then HIV drug resistance testing and the use of 3^rd^ line drugs, such as darunavir/ritonavir and raltegravir, amongst others, should be prioritised (Panel on antiretroviral guidelines for adults and adolescents and Department of Health and Human Services 2012; Panel on Antiretroviral Therapy and Medical Management of HIV Infected Children 2012). Identifying and addressing the cause of treatment failure in adolescents on boosted PI-based regimens will reduce the need for largely unavailable and expensive 3^rd^ line treatment.

This study sought to determine and quantify the causes of virological non-suppression, and determine if a home-based adherence intervention and standard care improved virological suppression in HIV-infected adolescents who are virologically failing atazanavir/ritonavir (ATV/r)-based 2^nd^ line treatment compared to standard care alone.

## METHODS

### Study design

A randomised, controlled trial (RCT) comparing modified directly administered antiretroviral therapy (mDAART) + standard care (SC) versus SC + self-administered treatment (SAT) for 90 days. Data was collected between January 2015 and May 2016. Eligible participants were included if they: were HIV positive with a documented result; were aged between 10 and 18 years; were on ATV/r-based 2^nd^ line treatment for ≥6 complete consecutive months; had virological treatment failure (viral load ≥1 000 copies/ml); knew their HIV status; provided informed consent and assent; were registered at Harare hospital paediatric opportunistic infections clinic and stayed within Harare hospital catchment area. Adolescents were consecutively screened for eligibility using a questionnaire and viral load measurement. The screening viral load was also used as baseline for enrolled participants. Participants were excluded if they were on anti-TB treatment; did not want to be followed-up at home; had viral load <1 000 copies/ml within the previous 2 months or were on ATV/r as 1^st^ line treatment.

Total patient sampling of eligible, assenting and consenting adolescents was considered after noting that the clinic had 267 children, adolescents and young adults on boosted protease inhibitors from 0 to 22 years of age, either as 1^st^ or 2^nd^ line treatment. The study was divided into 2 phases:

#### Phase 1:

Eligible participants were randomised to intervention (mDAART + SC) or control (SC + SAT) arms. Randomisation was done using random numbers sealed in opaque envelopes. Questionnaires were administered at baseline and after follow-up. Participants were followed for 90 days. At the end of follow-up, viral load was measured again. Self-reported adherence was measured using AIDS Clinical Trials Group (ACTG) adherence follow-up questionnaire (QLO702) and visual analogue scale (VAS) (Chesney et al., 2000; Walsh et al., 2002).

#### Phase 2:

**Figure 1.**
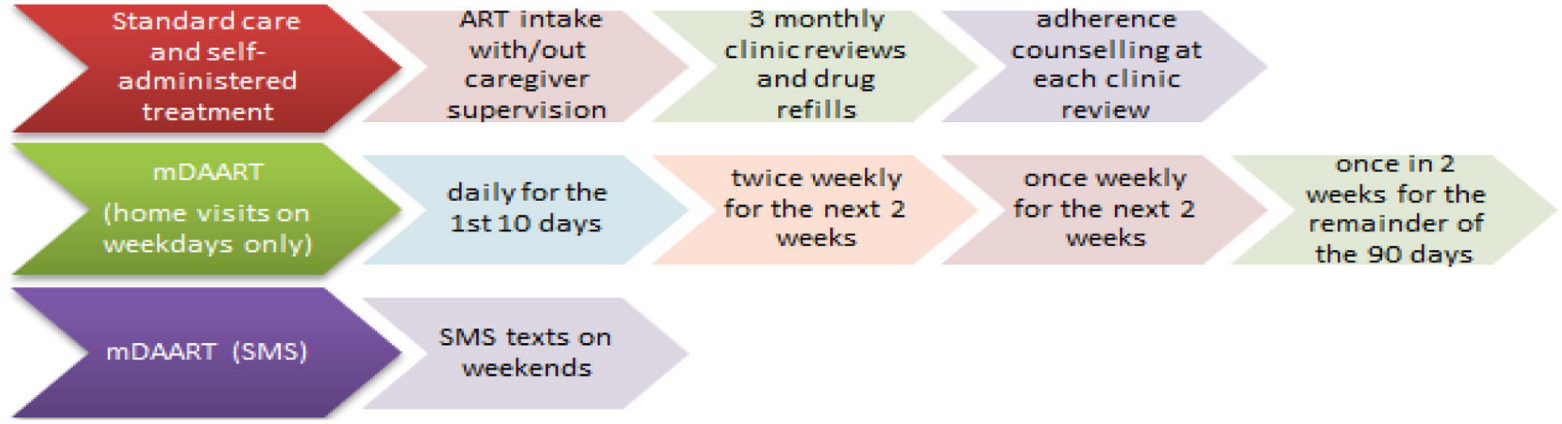
Components of study arms.

Participants with continued treatment failure (viral load ≥1 000 copies/ml) had genotypic HIV drug resistance testing. Components of each arm are summarised in Figure 1. Standard care (SC) consisted of 3 monthly hospital visits to see clinic doctors, adherence counselling by trained peer counsellors and drug refills at each hospital visit. SAT consisted of participants taking medication on their own, with or without supervision by caregivers. The intervention, mDAART, consisted of scheduled home visits during the week and short message service (SMS) on weekends by trained field workers. Home visits and SMS text messages were timed to coincide with the time participant was taking ATV/r. Home visits were scheduled during weekdays only (Mondays to Fridays) as shown in Figure 1. Trained field workers observed participants swallow medication and completed home visit charts. Participants were given a “pill chart” to complete over the 90 days.

Samples for viral load and HIV drug resistance testing were collected in 2×4 ml K-EDTA tubes respectively, gently inverted 8 to 10 times to prevent clotting, transported at atmospheric temperature to the laboratory. The Roche COBAS AmpliPrep/COBAS Taqman HIV-1 Test version 2.0 was used for viral load measurement, with a linear range of 20 to 10,000000 copies/ml. HIV drug resistance mutations were generated by the Celera ViroSeq® HIV-1 genotyping system version 2.0 (Abbott Molecular Diagnostics). Sequencing was done on 3500 Genetic Analyser supplied by Thermo Fisher, Life Technologies. Mutations were identified with ViroSeq software and analysed with the Stanford database (www.HIVDB.stanford.edu) to interpret drug susceptibility.

### Ethical approval

This study was approved by Harare hospital institutional review board, Joint Research Ethics Committee (JREC/51/14), Biomedical and Research Training Institute (BRTI) and Medical Research Council of Zimbabwe (MRCZ/A/1840). This clinical trial was registered with Pan African Clinical Trial Registry (PACTR201502001028169) and National Institutes of Health (NIH) Clinical Trials.gov ().

### Statistical considerations

Data from questionnaires was entered into research electronic data capture (REDCap), a web-based application (Harris et al., 2009). All data was analysed in Stata version 14 (Stata Corp). Treatment failure was defined as viral load ≥1 000 copies/ml after 90 days follow-up. We used Chi-square (and Fischer’s test where appropriate) and student’s t test to determine associations between mDAART, standard of care, self-reported adherence and virological suppression (<1 000 copies/ml. P-values are 2-sided and considered statistically significant if <0.05. Primary treatment outcome was defined as viral load <1 000 copies/ml after 90 days of follow-up.

Possible confounders and factors with p<0.25 in bivariate analysis were considered in multivariate analysis to adjust for the effect of mDAART on viral load and self-reported adherence including: age, gender, level of education, orphan and caregiver status; World Health Organisation (WHO) clinical stage at ART initiation; baseline, latest and on-treatment peak CD4 cell counts; time on 1^st^ line, 2 line and total time on ART; baseline, follow-up and change in viral load; pill burden per day; dosing frequency and body-mass index (BMI)-for-age (World Health Organisation Multicentre Growth Reference Study Group 2007). Stepwise logistic regression was used in multivariate analysis.

## RESULTS

**Figure 2.**
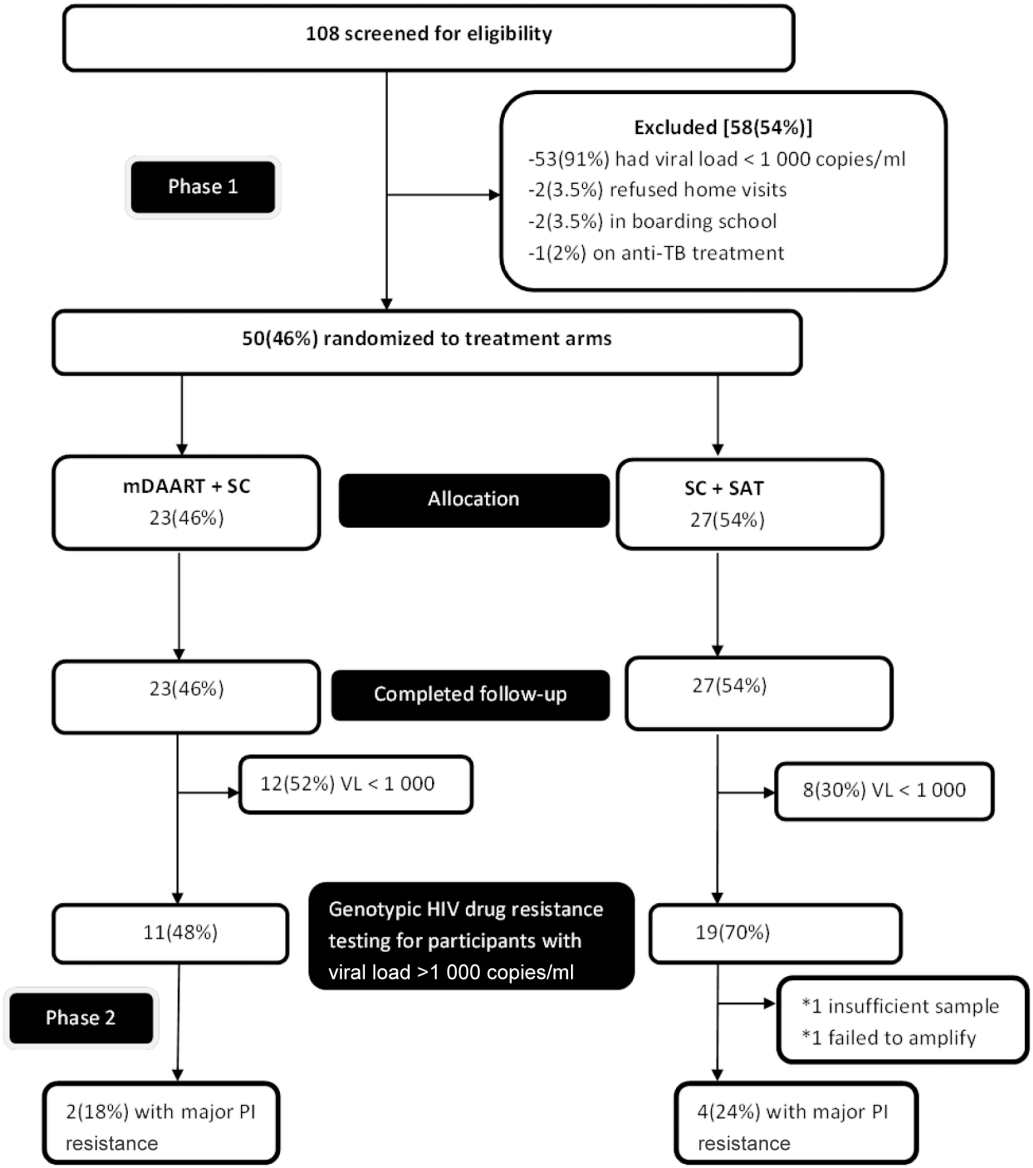
Consort flow chart of participants. *PI, protease inhibitor*.

Fifty participants were recruited. Of the participants who were screened, 53/108 (49%) were virologically suppressed (viral load <1 000 copies/ml). One hundred and six (98%) participants accepted home visits. Only 2/108 (2%) participants who were eligible refused home visits citing their intrusive nature. Twenty-three (46%) and 27(54%) participants were randomised to intervention and control arms respectively (Figure 2).

Mean age was 15.8 years. Most participants were either in secondary or high school (form 1–6) (78%). There were more females (54%) than males. 46% were double orphans. Only 20% lived with their biological parent(s). At initiation of 1^st^ line ART, 68% had WHO clinical stage 3 or 4 disease, and 42% had a CD4 cell count <200 cells/mm3. At enrollment into study, 52% had CD4 count <200 cells/mm3 and 30% had low BMI-for-age (thinness or severe thinness). Eighty-six percent were taking tenofovir/lamivudine (300 mg/300 mg) fixed dose combination (FDC) and ATV/r (300 mg/100 mg) FDC; 90% were taking a total of 2 to 4 ART tablets (including cotrimoxazole prophylaxis) a day; and 90% were taking ART (including cotrimoxazole prophylaxis) once a day. Mean total time on ART was 78 months (Table 1).

Treatment arms were well matched at baseline. Forty percent had average self-reported adherence <80% at baseline compared to 22% after follow-up, and 66% reported an increase in self-reported adherence after follow-up. Average self-reported adherence and ATV/r adherence by visual analogue scale were similar. Mean viral load change was −1.1 log_10_ copies/ml, 74% had overall decrease in viral load, 46% had ≥1 log_10_ decrease in viral load and 40% achieved virological suppression (viral load <1 000 copies/ml) (Table 2).

**Figure 3.**
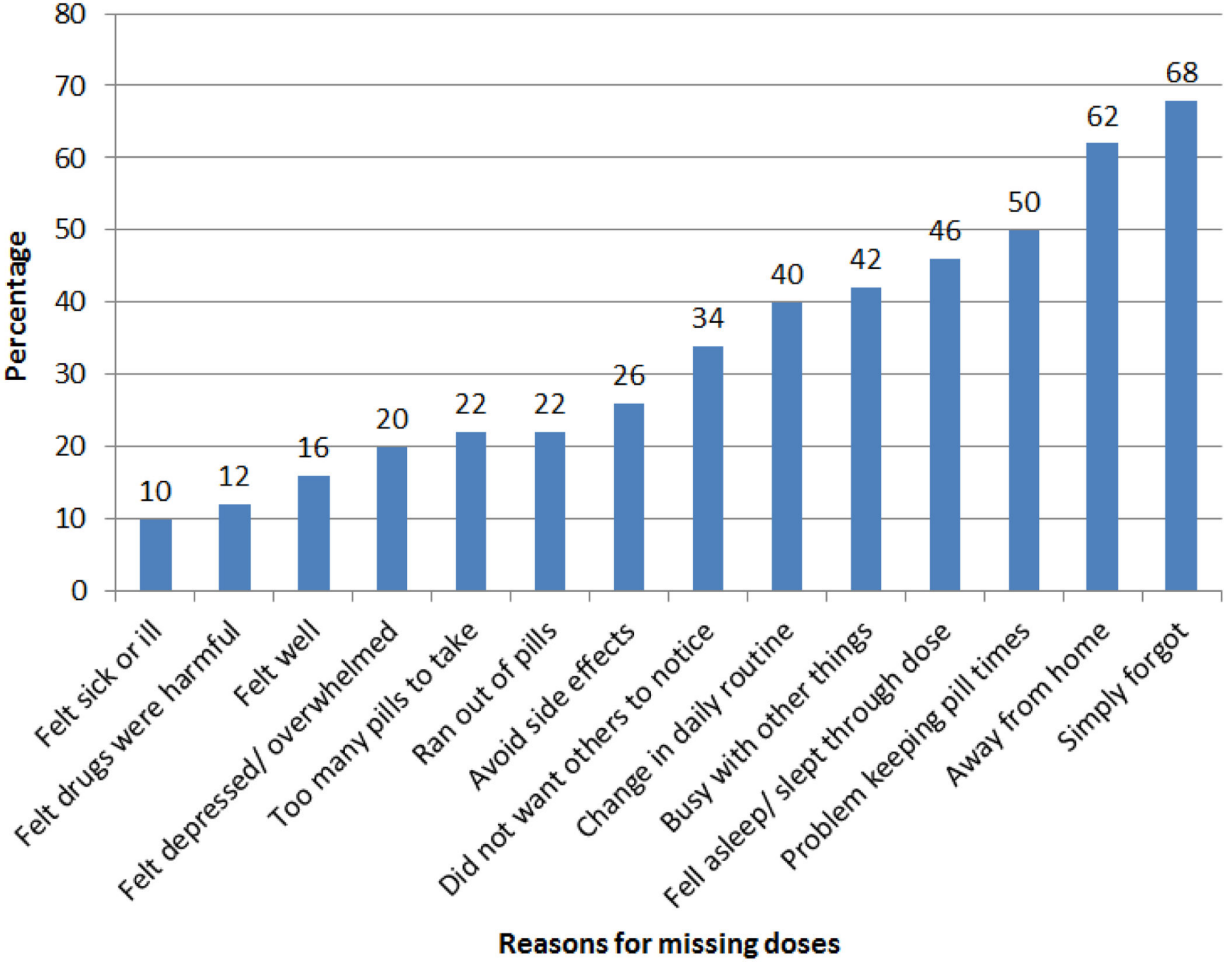
Reasons for missing ART doses.

Common reasons for missing ART were simply forgetting (68%), being away from home (62%), problem with keeping time (50%) and falling asleep before taking medication or waking up late (46%) (Figure 3).

52% of the participants in mDAART achieved virological suppression compared to 30% in standard care. There was a modest increase in viral load suppression in mDAART compared to SC after stratifying by viral load <1 000 vs ≥1 000 copies/ml (p=0.105). Viral load decreased more in mDAART arm compared to standard care (p=0.03) and viral load at follow-up was lower in mDAART compared to standard care (p=0.04). Average self-reported adherence in previous 1 month measured by visual analogue scale at follow-up was higher in mDAART compared to standard care (p=0.05), and the number of participants who reported closely following their dosing schedule in the previous 4 days was higher in mDAART compared to standard care at follow-up (p=<0.001) (Table 3).

There were no significant differences between suppressed and unsuppressed participants. Multivariate models were assessed comparing mDAART to SC, fitting self-reported adherence characteristics associated with virological suppression (Table 4).

Participants in mDAART were 90% less likely to report <80% adherence in the previous 1 month (p=0.023), were 4.8 times more likely to closely follow their dosing schedule in the previous 4 days (p=0.004) compared to those who were not exposed to the intervention (Table 5).

### Genotypic HIV drug resistance test

**Figure 4.**
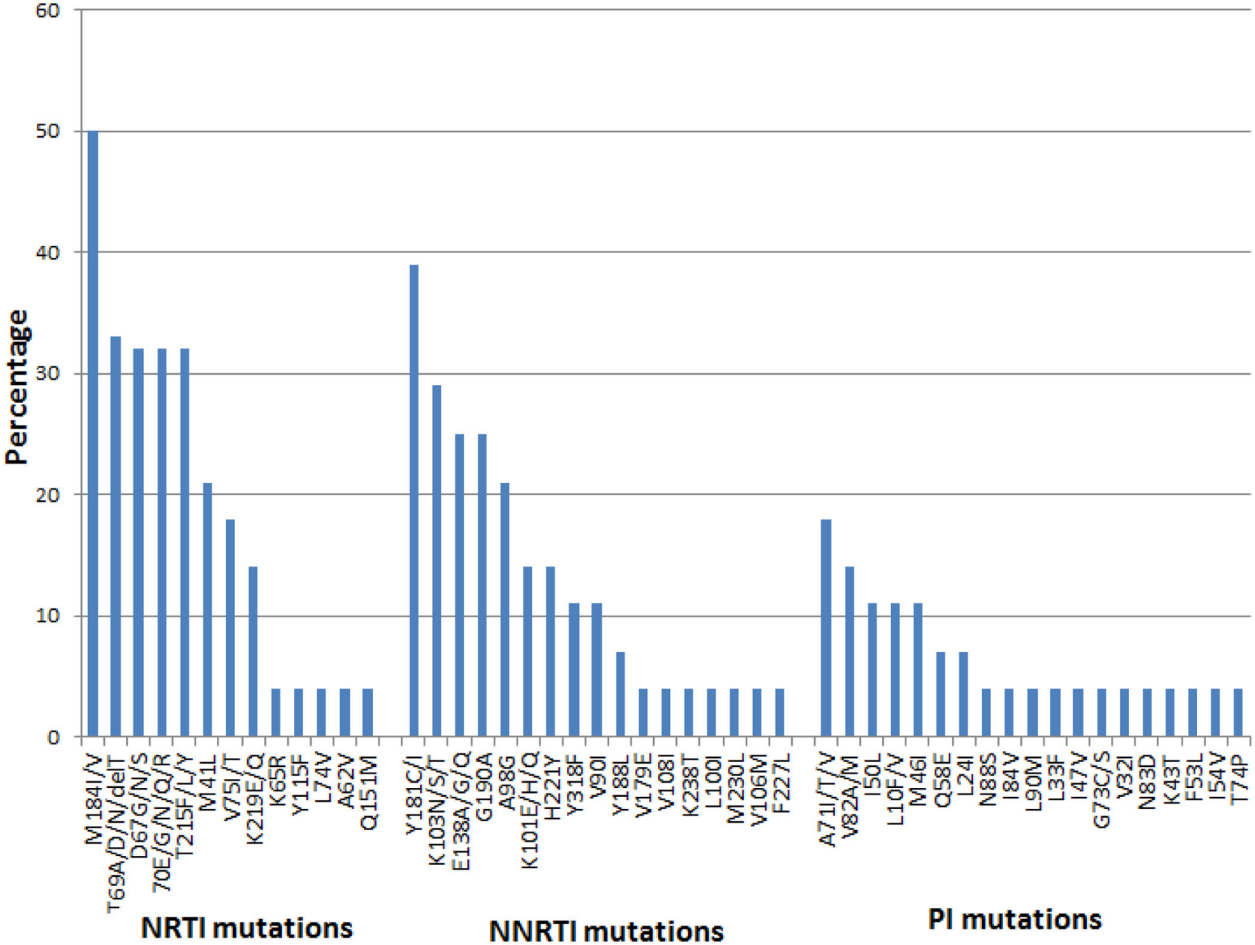
Frequency of HIV drug resistance mutations by ARV drug class.

Thirty (60%) participants had viral load >1 000 copies/ml at 3 months and 28/30 (93%) had a genotypic HIV drug resistance test within 1 month of follow-up viral load measurement. Three (11%) participants had wild type virus (Table 6). PI resistance was seen in 10(36%). High level atazanavir/ritonavir resistance was detected in 6(21%) of the 28 participants, 5 of whom had intermediate and/or low level ATV/r resistance mutations and 1 had a single I50L mutation. Three (11%) of the 4 participants with multiple PI resistance mutations had high level resistance to ATV/r, lopinavir/ritonavir (LPV/r) and darunavir/ritonavir (DRV/r) (V32I, I50L, I54V, I47V and V82A) and were switched to 3^rd^ line integrase strand transfer inhibitors (InSTI)-based regimens (raltegravir). The other 3 had no resistance to LPV/r, and were switched to LPV/r, which is the available alternative 2^nd^ line treatment (Table 6). The most frequent PI mutations were A71I/T/V (18%), V82A/M (14%), M46I (11%), L10F/V (11%) and I50L (11%) (Figure 4).

## DISCUSSION

Directly observed treatment (DOT) has been successfully implemented in anti-TB treatment. However, its use in HIV treatment is controversial. In our study, a short-term mDAART intervention provided to adolescents failing 2^nd^ line treatment was associated with a significantly greater decrease in viral load and increase in self-reported adherence compared to standard care, and it also modestly increased virological suppression. Our findings support earlier findings which found that DAART decreases viral load by an effect size between 20 and 30% and increases self-reported adherence when targeted to at-risk populations (Altice et al., 2004; Altice et al., 2007; Amico et al., 2006; Berg et al., 2011; Ford et al., 2009; Goggin et al., 2007; Lucas et al., 2006; Nachega et al., 2010; Wohl et al., 2006). At-risk groups include drug-abusers, patients with poorly controlled mental illness, homeless and marginally housed people.

We also found that 40% of adolescents had adherence <80% at baseline. Adolescent adherence to treatment is lower than that for children and adults (Kim et al., 2014; Sohn and Hazra, 2013). As children grow older, responsibility of HIV care usually shifts from caregiver to adolescent self-management (Modi et al., 2012; Taddeo et al., 2008). This transition usually coincides with complex psycho-social factors typical of this age group at a time of physical and emotional transition to adulthood (Davies et al., 2008; Lowenthal et al., 2014). Moreover, vertically infected adolescents are also likely to have been on ART for longer periods, resulting in treatment fatigue.

Forgetfulness was the most common cited reason for missing doses, and concurs with findings from earlier studies in adults (Barfod et al., 2006; Koole et al., 2016). mDAART allows direct observation of dose ingestion, reminding adolescents to take medication and providing psycho-social support. This increases adherence and decreases viral load if there is no drug resistance and drug exposure is adequate. Interestingly, among the common reasons for missing doses cited, there were no treatment related reasons. This finding is encouraging and supports earlier findings that ATV/r and tenofovir/lamivudine FDCs are tolerable due to favourable side effect profiles, once daily dosing and low pill burden (Achenbach et al., 2011; Dong et al., 2016; Wensing et al., 2010). This allows policy makers to concentrate on addressing psycho-social causes of non-adherence in adolescents.

Acceptance rate for home visits in our study was surprisingly higher than previously reported (Altice et al., 2007; Wohl et al., 2006). This finding is encouraging. Adolescents who are failing 2^nd^ line regimens are often going to school. A community or clinic-based DOT intervention could face challenges in implementation due to busy lifestyles and stigmatisation. A home-based adherence intervention offers lesser burden to adolescents. However, the cost involved in mDAART, the intrusive nature of the intervention, breach of confidentiality of HIV status and migration of participants pose challenges to implementation. If DAART is going to be implemented, there needs to be careful consideration to confidentiality of patients’ HIV status, convenience to the patient and flexibility. Community health workers can assume this responsibility as they are familiar with communities they work in and have a portfolio full of other responsibilities (contact tracing for TB, dysentery and other communicable diseases, and health awareness). Use of technology (SMS, automated calls, camera phones and video internet) could reduce the need for many physical home visits. Family members/friends could also observe dose ingestion on days that mDAART will not be done. Once daily ART regimens also ease implementation DAART.

Time on 2^nd^ line ART was shorter than time on 1^st^ line ART in this study. This finding concurs with findings from previous studies, and is worrying. Risk of subsequent treatment failure increases after 1^st^ line failure (Chawana et al., 2014). Adolescents that are failing 2^nd^ line ART are at high risk of failing 3^rd^ line and salvage regimens. Third line regimens are largely unavailable and where they are available, they require HIV drug resistance testing prior to switch to 3^rd^ line (Conradie et al., 2012; Federal Ministry of Health Nigeria, 2010; Ministry of Health Botswana, 2012; National Department of Health, 2012; World Health Organisation (WHO) 2010a; World Health Organisation (WHO) 2010b; World Health Organisation HIV/AIDS Programme, 2013). However, HIV genotypic drug resistance testing is unavailable in public health care in RSL, and is expensive in private laboratories (USD$382 and USD$795), which ship their samples to South Africa. Maintaining adequate adherence in adolescents could reduce the need for expensive 3^rd^ line treatment and HIV drug resistance testing.

Nearly one-fifth of participants demonstrated high level ATV/r resistance, and was the same as that found in adults (Boender et al., 2016). This finding contradicts previous studies which found that patients on boosted PIs who develop virological treatment failure do not have clinically significant PI mutations and they re-suppress after intensive adherence interventions (Garone et al., 2014; Levison et al,. 2012). Although ATV/r has high genetic barrier against resistance, perinatally infected adolescents often have long treatment histories, inconsistent treatment adherence and multi-drug experience resulting from numerous switches when treatment failure has occurred, all favouring evolution of drug resistance (MacDonell et al., 2013). This finding is extremely worrying due to limited supply of 3^rd^ line regimens in RSL. Beyond 2^nd^ line treatment, prognosis is poor. Persistence of high level NNRTI resistance in this study is also worrying because it rules out the possibility of future use of this drug class in the event that patients run out of treatment options.

## Conclusion

Administering a home-based DAART intervention with direct observation of dose ingestion and SMS reminders to adolescents who were failing 2^nd^ line treatment increased self-reported adherence and decreased viral load. High level PI resistance was also demonstrated. We recommend that HIV drug resistance testing and 3^rd^ line antiretroviral treatment, like darunavir/ritonavir and raltegravir, be made more available in RSL in anticipation of a surge in PI resistance. We also propose that HIV drug resistance testing be done at time of diagnosis of 2^nd^ line treatment failure. Waiting 3 to 6 months for a 2^nd^ viral load results in disease progression and creates a window for spread of PI resistant virus.

### Limitations

The mDAART intervention was based on SMS reminders and observation of dose ingestion. It is therefore difficult to separate the effect of each component. Future studies could separate these 2 components and compare their effects individually. In addition, frequency of home visits were intensive at the beginning and reduced with time, therefore their effect might have waned off as the visits reduced. The intervention was administered for 3 months, which is relatively short. There was no follow-up after the intervention was discontinued to see if the effect of mDAART would be sustained. Measurement of adherence using self-reports is known to overestimate adherence due to recall bias and social desirability. Even in the presence of adequate adherence, drug exposure may be inadequate (such as in chronic gastroenteritis and increased drug clearance in enzyme induction), resulting in treatment failure. Our sample was also small for power to be adequate.

## Figures and Tables

**Table 1.** Baseline socio-demographic and treatment characteristics.

Variable	Result (n=50) n(%) or mean(SD); 95% CI
Age (years)	15.8 (1.8); 11 – 18
Gender	
Female	27(54)
Male	23(46)
Current level of education	
Primary	4(8)
Secondary/advanced	39(78)
Other	7(14)
Orphan status	
Non-orphan (both parents alive)	7(14)
Single orphan	20(40)
Double orphan	23(46)
Caregiver	
Parent/s	10(20)
Other (grandparent/s, sibling, aunt/uncle)	40(80)
WHO clinical stage at ART initiation	
1–2	16(32)
3–4	34(68)
CD4 cell count at ART initiation (cells/mm^3^)	
<200	21(42)
200–350	9(18)
>350	20(40)
CD4 cell count at enrollment (cells/mm^3^)	
<200	26(52)
200–350	12(24)
>350	12(24)
On-treatment peak CD4 cell count (cells/mm^3^)	
<200	2(4)
200–350	4(8)
>350	44(88)
Basis of diagnosis of 1^st^ line treatment failure	
Clinical	33(66)
Immunological	47(94)
Virological	28(56)
Time on 1^st^ line ART (months)	55(26); 6–107
Time on 2^nd^ line ART (months)	22(10); 8–66
Total time on ART (months)	78(26); 24–134
Current treatment	
Tenofovir/lamivudine/atazanavir/ritonavir	43(86)
Zidovudine/lamivudine/atazanavir/ritonavir	3(6)
Abacavir/lamivudine/atazanavir/ritonavir	2(4)
Abacavir/didanosine/atazanavir/ritonavir	2(4)
Cotrimoxazole prophylaxis	49(98)
Pill burden per day	
2–4	45(90)
5–6	5(10)
Dosing frequency per day	
Once daily	45(90)
Twice daily	5(10)
BMI-for-age	
Underweight (severe thinness and thinness)	14(30)
Normal	25(55)
Overweight	7(15)

WHO = World Health Organization; ART= antiretroviral therapy; BMI = body mass index.

**Table 2.** Treatment characteristics at baseline and after follow-up.

Variable	Baseline (n=50) n(%) or mean(SD); 95% CI	After follow-up (n=50) n(%) or mean(SD); 95% CI
Average self-reported adherence, VAS (%)		
≥95	15(30)	25(50)
80–94	15(30)	14(28)
<80	20(40)	11(22)
ATV/r self-reported adherence, VAS (%)		
≥95	16(32)	28(56)
80–94	15(30)	11(22)
<80	19(38)	11(22)
Change in average self-reported adherence, VAS:		
No change	-	7(14)
Decreased	-	10(20)
Increased	-	33(66)
Missed all doses in a day in past 4 days		
Yes	15(30)	5(10)
No	35(70)	45(90)
Missed at least 1 dose in past 4 days		
Yes	18(36)	18(36)
No	32(64)	32(64)
Closely followed dosing schedule in past 4 days		
Yes	22(44)	29(58)
No	28(56)	21(42)
Missed at least 1 dose previous weekend		
Yes	12(24)	12(24)
No	38(76)	38(76)
Last time a dose/s was missed		
0–4 weeks ago	28(56)	18(36)
>4 weeks ago	22(44)	32(64)
Viral load (log _10_ copies/ml)	4.8(0.8); 3–7	3.7(1.5); 1.3–5.9
Viral load change (log_10_ copies/ml)	-	−1.1(1.5); −5.5–2
Viral load change:		
Decreased	-	37(74)
Increased	-	13(26)
≥1 log_10_ decrease in viral load	-	23(46)
<1 log_10_ decrease in viral load	-	27(54)
Viral load, copies/ml		
<1 000	-	20(40)
≥1 000		30(60)

VAS, *visual analogue scale; ATV/r, atazanavir/ritonavir*.

**Table 3.** Comparison of participants’ treatment characteristics by treatment arms.

Variable	mDAART (n=23) n(%) or mean(SD); 95% CI	Standard care (n=27) n(%) or mean(SD); 95% CI	p-value
Viral load at follow-up			
<1 000 copies/ml	12(52)	8(30)	0.105
≥1 000 copies/ml	11(48)	19(70)	
Viral load change			
≥1 log_10_ decrease	12(52)	11(41)	0.399
<1 log_10_ decrease	11(48)	16(59)	
Follow-up viral load (log_10_ copies/ml)	3.3(1.5); 2.6–3.9	4(1.5); 3.4–4.6	0.048
Viral load decrease (log_10_ copies/ml)	−1.5(1.6); −2.2– −0.9	−0.8(1.3); −1.3– −0.3	0.031
Average self-reported adherence, (VAS) at follow-up (%)			
≥95	15(65)	10(37)	
80–94	6(26)	8(30)	0.050
<80	2(9)	9(33)	
Change in average self-reported adherence (VAS)			
No change	3(13)	4(15)	
Increased	17(74)	16(59)	0.538
Decreased	3(13)	7(26)	
Missed all doses in a day in past 4 days at follow-up			
Yes	1(4)	4(15)	0.357
No	22(96)	23(85)	
Missed at least 1 dose in past 4 days			
Yes	2(9)	7(26)	0.114
No	21(91)	20(74)	
Closely followed dosing schedule in past 4 days at follow-up			
Yes	19(83)	10(37)	<0 001
No	4(17)	17(63)	
Missed at least 1 dose in previous weekend at follow-up			
Yes	3(13)	3(11)	0.985
No	20(87)	24(89)	
Last time a dose was missed at follow-up			
0–4 weeks ago	7(30)	11(41)	0.449
>4 weeks ago	16(70)	16(59)	

VAS, visual analogue scale.

**Table 4.** Comparison by viral load suppression to <1 000 copies/ml after 3 months.

Variable	Viral load <1 000 copies/ml (n=20) n(%) or mean(SD); 95% CI	Viral load ≥1 000 copies/ml (n=30) n(%)or mean(SD); 95% CI	p-value
Age (years)	15(1.98); 14.4–16.3	16(1.66); 15.4–16.7	0.08
Gender:			
Female	10(50)	17(57)	0.643
Male	10(50)	13(43)	
Current level of education			
Primary	2(11)	2(8)	
Secondary/advanced	15(83)	24(92)	0.582
Other	1(6)	0(0)	
Orphan status:			
None	2(10)	5(17)	
Single orphan	8(40)	12(40)	0.858
Double orphan	10(50)	13(43)	
Caregiver:			
Parent/s	3(15)	7(23)	0.470
Other (grandparent/s, sibling, aunt/uncle)	17(85)	23(77)	
WHO clinical stage at ART initiation			
1–2	8(40)	8(27)	0.322
3–4	12(60)	22(73)	
CD4 cell count at ART initiation (cells/mm^3^)			
<200	8(40)	13(43)	
200–350	5(25)	4(13)	0.563
>350	7(35)	13(43)	
CD4 cell count at enrollment (cells/mm^3^)			
<200	7(35)	19(63)	
200–350	6(30)	6(20)	0.133
>350	7(35)	5(17)	
On treatment peak CD4 cell count (cells/mm^3^)			
<200	0(0)	2(7)	
200–350	2(10)	2(7)	0.650
>350	18(90)	26(86)	
Time on 1^st^ line ART (months)	57.3(18.6); 48–62	52.8(30); 41–64	0.281
Time on 2^nd^ line ART (months)	21.8(8.3); 17.8–25.9	22.5(11); 18.3–26.7	0.409
Total time on ART (months)	81.3(17.6); 73–90	75.3(30.8); 63–87	0.217
Dosing frequency per day at follow-up			
Once daily	19(95)	28(93)	1.000
Twice daily	1(5)	2(7)	
BMI-for-age			
Normal	12(63)	13(48)	
Underweight (severe thinness and thinness)	4(21)	10(37)	0.499
Overweight	3(16)	4(15)	
Treatment arm			
mDAART	12(60)	11(37)	0.105
Standard care	8(40)	19(63)	
Average self-reported adherence, (VAS) at follow-up (%)			
≥95	10(50)	15(50)	
80–94	8(40)	6(20)	0.143
<80	2(10)	9(30)	
Change in self-reported adherence (VAS)			
No change	5(25)	2(7)	
Increased	11(55)	22(73)	0.181
Decreased	4(20)	6(20)	
Missed all doses in a day in past 4 days at follow-up			
Yes	1(5)	4(13)	0.636
No	19(95)	26(87)	
Missed at least 1 dose in past 4 days			
Yes	3(15)	6(20)	0.652
No	17(85)	24(80)	
Closely followed dosing schedule in past 4 days at			
follow-up			
Yes	14(70)	15(50)	0.160
No	6(30)	15(50)	
Missed at least 1 dose in previous weekend at follow-up			
Yes	3(15)	3(10)	0.672
No	17(85)	27(90)	
Last time a dose was missed at follow-up			
0–4 weeks ago	7(35)	11(37)	0.904
>4 weeks ago	13(65)	19(63)	

mDAART, modified directly administered antiretroviral therapy; VAS, visual analogue scale.

**Table 5.** Multivariate logistic regression comparing mDAART referenced to standard care.

Variable	Relative risk (95% confidence interval)	p Value
Average self-reported adherence, (VAS) at follow-up (%)		
≥95	-	-
80–94	0.4(0.1–1.5)	0.162
<80	0.1(0.02–0.8)	0.023
Closely followed dosing schedule in past 4 days at follow-up		
No	-	-
Yes	4.8(1.6–13.8)	0.004

**Table 6.** Resistance mutations by ARV drug class.

Participant	Protease inhibitor mutations	NRTI mutations	NNRTI mutations
1	L10F, *M46I*, Q58E, A71I, I84V*	M41L, D67G, T69N, K70N, V75I, M184V*, T215F	A98G, *V179E*, Y181C*, G190A*
2	I50L*	M41L, D67G, V75I, M184V/I*, K70Q, T215F	Y188L*
3	Q58E, V82M	D67G, M184V*, T69D, K70R, K219Q	A98G, Y181C*, G190A*, K101E
4	-	D67G, K70R, T215I, T219E	G190A*, E138G
5	-	-	A98G, Y181C*, V90I
6	-	-	-
7	-	-	*K101H/Q*
8	-	M184V*	K103N*, *E138A*
9	-		
10	-	D67G, M184V*, K70G	A98G, Y318F
11	-	T69N	Y181C*, G190A*, K101E, V90I
12	-	-	Y181C*, K103T, H221Y
13	-	V75I, M184V*, K65R*, D67N, Y115F, K219E	Y181C*, V108I
14	A71T	M41L, T69N, K70R, D67N, T215L, K219E	A98G, Y181C*, K103N*, K238T
15	-	M184V*, K70E/G/R, D67N	V90I, K103N*, Y318F
16	-	M184I/V*	G190A*
17	*L90M*	T69N	-
18	-	M41L, M184V*, T215C/Y	K103N*, Y318F, E138Q
19	-	M41L, V75I, M184V*, T215F/Y	*E138A*, H221Y, Y181I
20	A71I/T, N88S*, L10V	T69A/N, M184V*, T215F, K70R, K219E, D67S, L74V	K103N*, L100I*, M230L*
21	_	T69D/N	Y181C*, G190A*
22	-	T69N	Y181C*
23		-	-
24	*M46I*, I50L*, L10V, L33F, *I47V*, A71V, G73C/S, V82A^#^	M184V*, T215F	A98G, G190A*, K101E, *E138A*
25	*M46I*	-	Y181C*, E138G, H221Y
26	I50L*, V82M, V32I^#^, L24I, N83D	M41L, K70N, V75I, M184V*, T215Y	H221Y, K103S*, V106M*, F227L^#^
27	V82M, A71V, L24I, K43T, F53L, I54V^#^, T74P	M184V*, A62V, 69deIT, V75T, Q151M	Y188L*
28	-	-	K103N*, *E138A*
Without mutations, n(%)	18(64)	8(29)	4(14%)

* high level resistance;

# intermediate level resistance;

Italics-low level resistance; PI, protease inhibitor; NNRTI, non-nucleotide reverse transcriptase inhibitors; NRTI, nucleot/side reverse transcriptase inhibitors.
